# Influence of international co-authorship on the research citation impact of young universities

**DOI:** 10.1007/s11192-016-1905-6

**Published:** 2016-03-15

**Authors:** K. A. Khor, L.-G. Yu

**Affiliations:** Research Support Office and Bibliometrics Analysis, Nanyang Technological University, #B4-01, Block N2.1, 76, Nanyang Drive, Singapore, 637331 Singapore

**Keywords:** International collaboration, International co-authorship, Young universities, Old universities, citations per paper (CPP), World university ranking, Field-weighted citation impact (FWCI), Research expenditure, Leiden ranking

## Abstract

We investigated the effect of international collaboration (in the form of international co-authorship) on the impact of publications of young universities (<50 years old), and compared to that of renowned old universities (>100 years old). The following impact indicators are used in this study, they are: (1) the 5-year citations per paper (CPP) data, (2) the international co-authorship rate, (3) the CPP differential between publications with and without international co-authorships, and (4) the difference between the percentage of international co-authored publications falling in the global top 10 % highly cited publications and the percentage of overall publications falling in the global top 10 % highly cited publications (Δ%_Top10%_). The increment of 5-year (2010–2014) field weighted citation impact (FWCI) of internationally co-authored papers over the 5-year overall FWCI of the institutions in SciVal^®^ is used as another indicator to eliminate the effect of discipline difference in citation rate. The results show that, for most top institutions, the difference between the citations per paper (CPP) for their publications with and without international co-authorship is positive, with increase of up to 5.0 citations per paper over the period 1996–2003. Yet, for some Asian institutions, by attracting a lot of researchers with international background and making these collaborating “external” authors as internal researchers, these institutions have created a special kind of international collaboration that are not expressed in co-authorship, and the CPP gaps between publications with and without international co-authorship are relatively small (around 0–1 citations per paper increment) for these institutions. The top old institutions have higher CPP than young institutions, and higher annual research expenditures; while young universities have a higher relative CPP increment for the current 5-year period over the previous 5-year period. The Δ%_Top10%_ for international co-authored publications is generally higher than that for all journal publications of the same institution. With the increase of international co-authorship ratio, the mean geographical collaboration distance (MGCD, an indication of increased international co-authorship) of one institution based on the Leiden Ranking data also increases, and young institutions have relatively higher CPP increment over MGCD increment. International co-authorship has a positive contribution to the FWCI of the institution, yet there are untapped potential to enhance the collaboration among young institutions.

## Introduction

It is widely presumed that research collaboration; especially international collaboration, has benefits for both the researchers and the organisations involved, and enhances the quality of research (Katz and Hicks 1997; Van Raan 1998; Leydesdorff and Wagner 2008; Persson 2010; Van den Besselaar et al. 2012), resulting in higher numbers of scholarly output (publications) and higher impacts (citations) (Glanzel et al. 1999; Glanzel and Schubert 2001; Glanzel 2001; Hara et al. 2003; Persson et al. 2004). Through collaboration, partners can share costs and resources, access high-end facilities, synergise each other’s expertise, share newly developed techniques, skills, and knowledge, fortify areas of deficiencies and gain through diversity in professional cultures. Collaborative activity can add value and offer insights to key issues of concern, and address transnational or global problems.

**Fig. 1:**
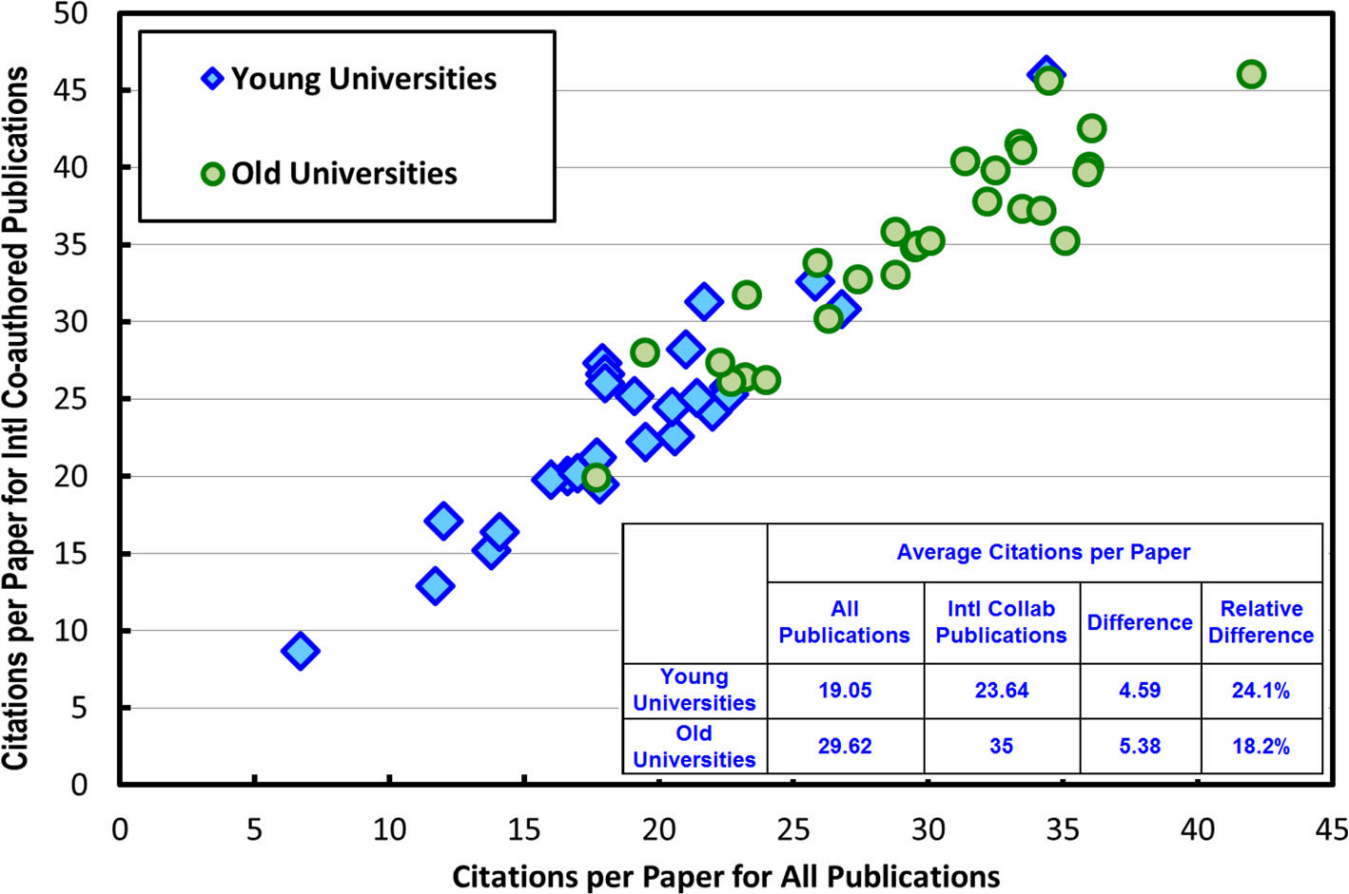
CPP for internationally co-authored publications against CPP for all publications for top young and old institutions in the Times Higher Education 2014 Ranking. (*Source*: Elsevier SciVal, *Data Set*: Collaboration Impact versus Citations per Publication; Data Range 1996–2013)

Figure 1 shows a plot of the CPP of internationally collaborated publications in the form of international co-authorship against the citations per paper (CPP) of all publications (Source: Elsevier SciVal; period: 1996–2013) for the top 26 young universities (<50 years old) and top 28 old universities (>100 years old) ranked by the Times Higher Education 2014 Ranking. It shows that international co-authorship is generally beneficial for institutions. The CPP for international co-authored publications have an average increment of 4.59 over CPP for all publications for young universities, and that for old universities is 5.38. It should be noted that the results presented in Fig. 1 may suffer from a field bias due to differences in citation rate among disciplines. That’s to say, if an institution has fields involving more international co-authorships with high citation frequencies, the CPP increment ratio for that institution may be relatively high. In view of this, some bibliometrics analysis databases have developed special impact indicators to address this field bias, like the category normalized citation impact (CNCI) in Thomson Reuters InCites™ database, and field-weighted citation impact (FWCI) in the Elsevier SciVal database. It also should be noted that, the results may depend on the types of collaborations, specifically, whether it is bilateral collaboration or multilateral collaboration (De Lange and Glänzel 1997; Glänzel and De Lange 1997). In this paper, we will also discuss the impact of international co-authorship expressed by FWCI, and the different impact of multilateral collaboration and bilateral collaboration in detail.

In recognizing the positive contribution of international co-authorship to an institution, several institutional ranking systems adopt indicators for it, for example, the International Outlook in the Times Higher Education World University Ranking (THE World University Ranking 2013/2014), and collaboration indicators in the Leiden Ranking (CWTS Leiden Ranking 2013, 2014). In the 2013 Leiden Ranking, 4 indicators are used as collaboration indicators: PP(collab)—proportion of inter-institutional collaborative publications; PP(int collab)—proportion of international collaborative publications; PP(UI collab)—proportion of collaborative publications with industry; and MGCD—mean geographical collaboration distance (CWTS Leiden Ranking 2013 & before; Waltman et al. 2012). MGCD is a unique indictor of collaboration in the Leiden Ranking (CWTS Leiden Ranking 2013; Waltman et al. 2012).

However, research also suggests that the effects of international collaboration may vary across disciplines and the authors’ countries (Moed 2005a). Scholars in developing nations especially favour international collaboration, as their internationally collaborated papers will be more visible and more frequently cited in prestigious journals than their traditional papers without international collaborations (Cronin and Shaw 1999). This finding inspired the research question for this study: is international collaboration in the form of international co-authorship a beneficiary for young and ambitious institutions? The answer is unclear, as research on international co-authorship of young institutions is not extensively studied. On the other hand, Moed (2005a) also argued that the effect of international collaboration also depends upon how the collaboration is organized, and which institution or country takes the lead in the collaboration, and which group plays a more secondary role. They found that, when a high and a low citation impact country collaborated, their order is indeed significant. When the former came first, and hence delivered the primary author or leading research group, 67 % of collaboration pairs produced bi-lateral international collaboration papers with an average citation impact above the mean citation impact of purely domestic papers from the two. But when a low impact country came first, this percentage dropped to 43. This notion is embodied in a new indicator developed by Moya-Anegon et al. (2013), denoted as “research guarantor” indicator. It is expected that effect of international co-authorship of the young and ambitious institutions also depends on how the collaboration is organized. Yet, we will not address this point in detail in this study.

In this study, we investigated the effect of international co-authorship on the impact of publications of top young universities, and compared to that of some old universities. The 5-year CPP data, the international co-authorship rate, the CPP difference between publications with and without international co-authorships, and the difference between the percentages of international co-authored publications falling in the global top 10 % highly cited publications and the percentage of overall publications falling in the global top 10 % highly cited publications (Δ%_Top10%_) are used as the impact indications. These data are extracted from the Thomson Reuters Web of Science (WoS) database and Essential Science Indicator (ESI) based on papers published from 2004 to 2013. The mean geographical collaboration distance (MGCD) as a function of PP(int collab) (proportion of international collaborative publications) in the Leiden Ranking (CWTS Leiden Ranking 2011/2012, 2013) were plotted to reveal the geographical effect of scientific collaboration. Young institutions ranked by the 2014 Times Higher Education (THE)’s 100 under 50 Universities are selected in this study, and some renowned universities (>100 years old) are selected as references for “old universities”.

To eliminate the discipline difference effect, the increment of 5-year (2010–2014) field weighted citation impact (FWCI) of internationally co-authored papers over the 5-year overall FWCI of the institutions in SciVal^®^ of Elsevier is used as another indicator, and the collaboration among some selected old institutions and young institutions are investigated.

## Method and data

The Thomson Reuters Web of Science database is used to extract the publication data, and the international co-authorship rate was calculated from the number of international collaborating publications therein. The 5-year CPP data for All Fields in the Thomson Reuters Essential Science Indicators is used as one of the impact indicators. The 5-year CPP difference between the publications with and without international co-authorships (ΔCPP) are calculated from the publication data, and the difference between the percentages of international collaborated publications falling in the global top 10 % highly cited publications and the percentage of overall publications falling in the global top 10 % highly cited publications (Δ%_Top10%_) are the other two indicators of impact. The analysis is based on papers published from 2004 to 2013. Only publications in the Web of Science Core Collection: Citation Index Expanded (SCI-Expanded), Social Sciences Citation Index (SSCI) and Arts & Humanities Citation Index (A&HCI) are used for publication search.

The mean geographical collaboration distance (MGCD) and PP(int collab) (proportion of international collaborative publications) in the Leiden Ranking (CWTS Leiden Ranking, 2011/2012, 2013) were used for plotting the MGCD versus PP(int collab) graph. MS Excel is used for data analysis in obtaining the bibliometric indicators and plotting the graphs for the study.

The 5-year field weighted citation impact (FWCI) of internationally co-authored publications and the 5-year overall FWCI of the collaborating institutions are extracted from SciVal^®^ of Elsevier to investigate the impact of international co-authorship to the FWCI of the institutions.

## Results and discussion

### Correlation between International co-authorship rate and CPP in 5-year interval

**Fig. 2:**
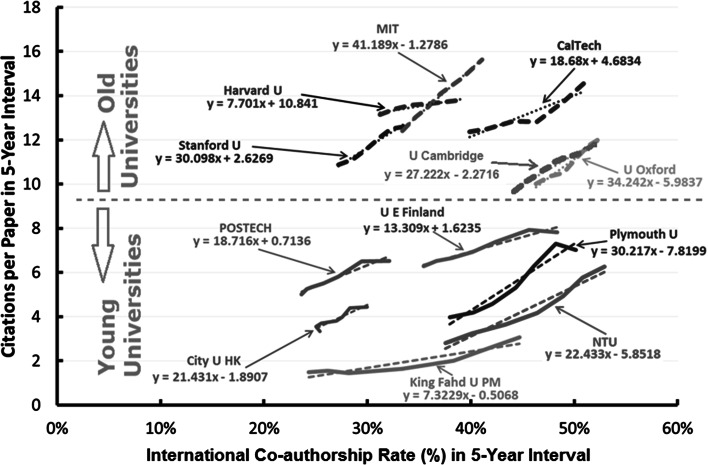
5-year CPP trends versus 5-year international co-authorship rate trends for selected young and old universities. *MIT* Massachusetts Institute of Technology (USA), *Harvard U* Harvard University (USA), *Stanford U* Stanford University (USA), *CalTech* California Institute of Technology (USA), *U Oxford* University of Oxford (UK), *POSTECH* Pohang University of Science And Technology (South Korea), *City U HK* City University of Hong Kong, *U E Finland*, University of Eastern Finland, *NTU* Nanyang Technological University (Singapore), *Plymouth U* Plymouth University (UK), *King Fahd U PM* King Fahd University of Petroleum and Minerals (Saudi Arabia)

**Fig. 3:**
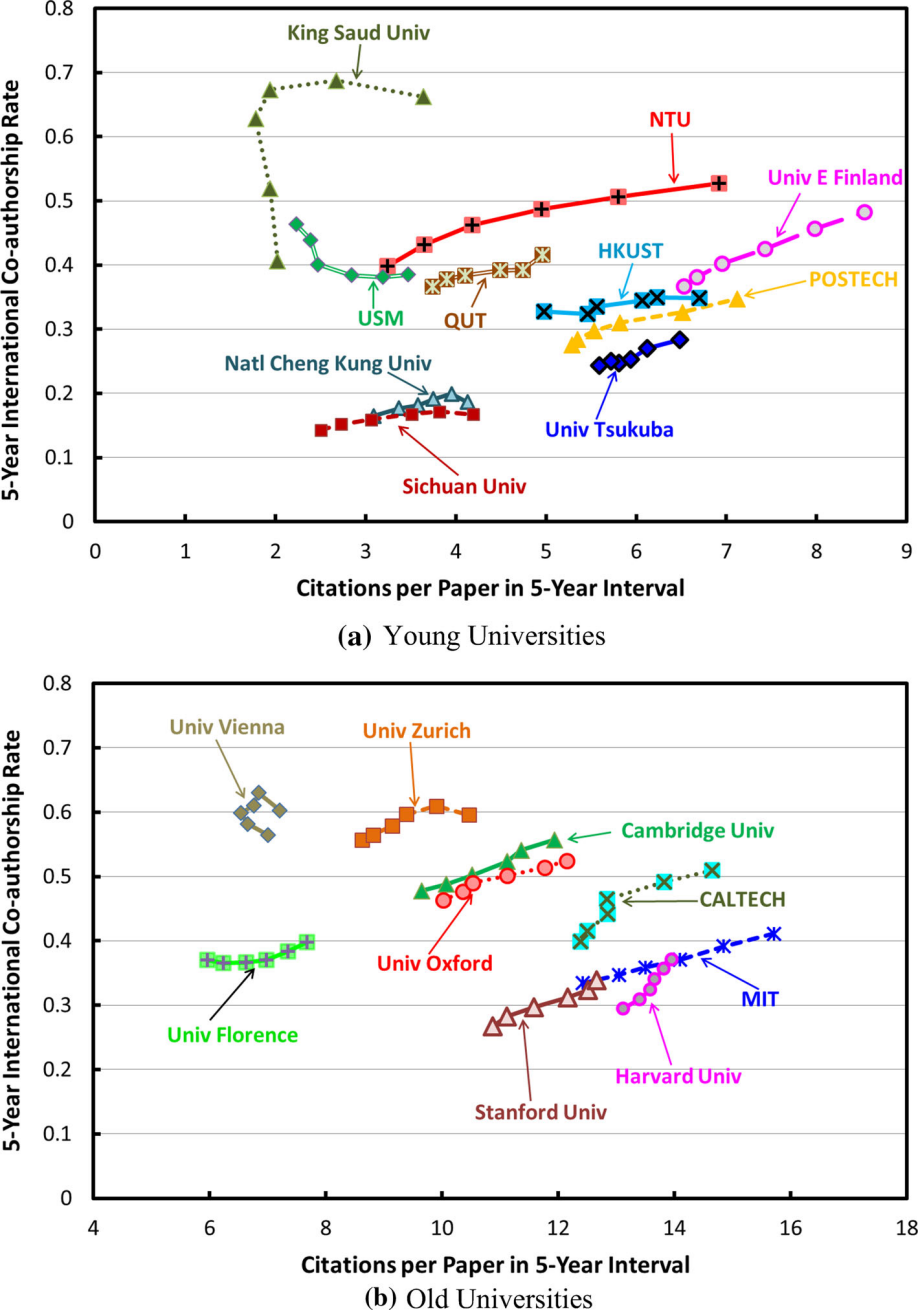
5-year international co-authorships rate trends versus 5-year CPP trends for selected **a** young and **b** old universities. *King Saud Univ* King Saud University (Saudi Arabia), *NTU* Nanyang Technological University (Singapore), *USM* Universiti Sains Malaysia (Malaysia), *QUT* Queensland University of Technology (Australia), *HKUST* Hong Kong University of Science and Technology, *U E Finland* University of Eastern Finland, *POSTECH* Pohang University of Science and Technology (South Korea), *Natl Cheng Kung Univ* National Cheng Kung University (Taiwan), *Univ Tsukuba* University of Tsukuba (Japan), *Sichuan Univ* Sichuan University (PR China), *Univ Vienna* University of Vienna (Austria), *Univ Zurich* University of Zurich (Switzerland), *Univ Florence* University of Florence (Italy), *MIT* Massachusetts Institute of Technology (USA), *Harvard U* Harvard University (USA), *Stanford U* Stanford University (USA), *CalTech* California Institute of Technology (USA), *U Oxford* University of Oxford (UK), *Cambridge Univ* Cambridge University (UK)

Figures 2 and 3 show the 5-year ESI CPP trends as a function of 5-year international co-authorships rate trends for selected young and old universities. While old universities have higher CPP in general, there are strong correlation between international co-authorship rate trends and 5-year CPP trends. For example, the CPP increased 4.12 cites for every 10 % increase in international co-authorship rate for Massachusetts Institute of Technology (MIT, USA), 3.42 cites for University of Oxford (U Oxford, UK), and 3.01 cites for Stanford University (Stanford U, USA). Among young universities, for Nanyang Technological University (NTU, Singapore), it is 2.24 cites per 10 % Intl Collab increment, and that for Plymouth University (Plymouth U, UK) is 3.02 cites, and 0.73 cites for King Fahd University of Petroleum and Minerals (King Fahd U PM, Saudi Arabia).

**Fig. 4:**
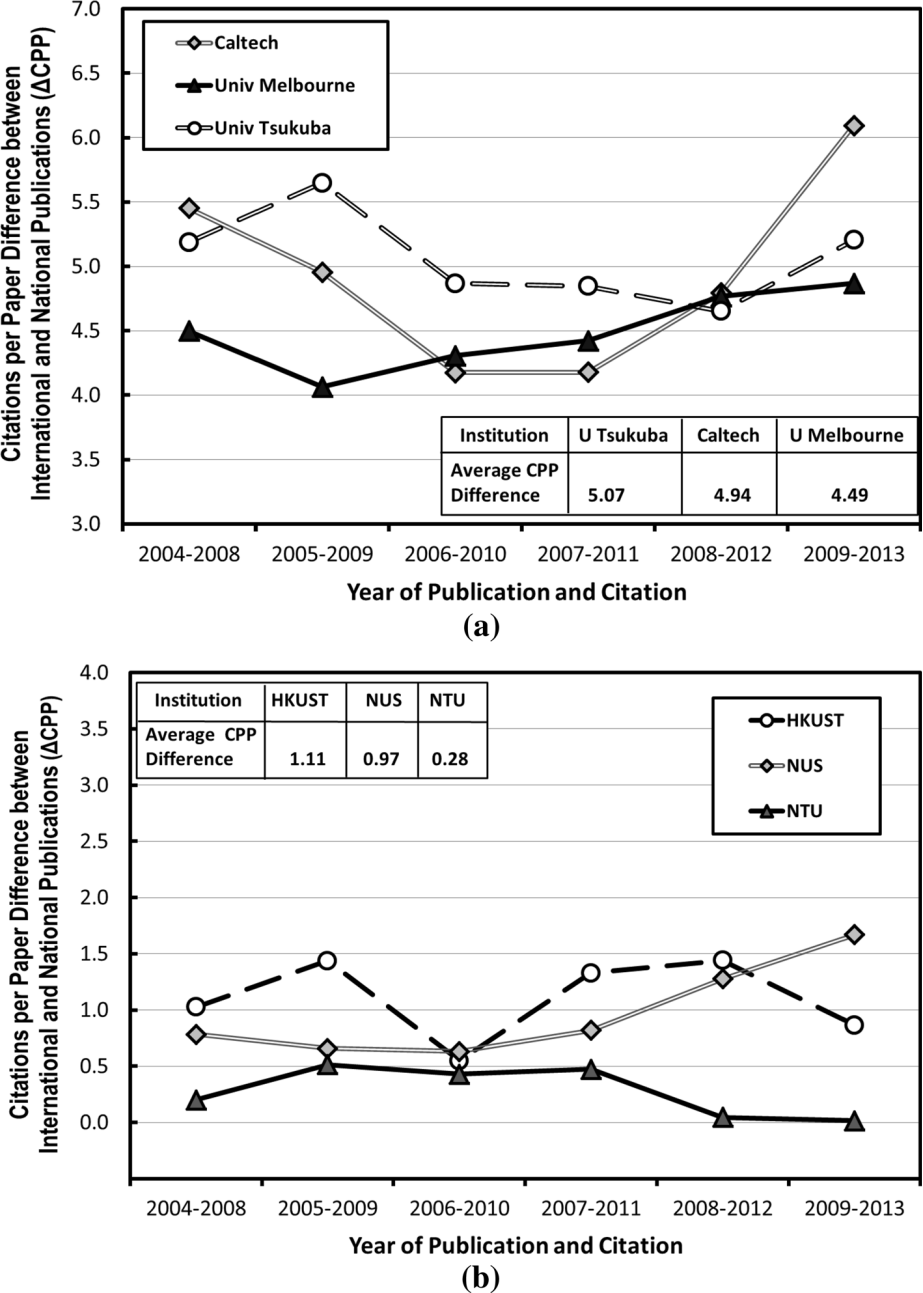
5-year CPP trends for publications with and without international co-authorships for **a** Caltech, U Tsukuba and U Melbourne, and **b** HKUST, NUS and NTU. *CalTech* California Institute of Technology (USA), *U Tsukuba* University of Tsukuba (Japan), *U Melbourne* University of Melbourne (Australia), *HKUST* Hong Kong University of Science and Technology, *NUS* National University of Singapore, *NTU* Nanyang Technological University (Singapore)

Figure 4 shows the ΔCPP trends for publications with and without international co-authorships for (a) Caltech, Univ Tsukuba and Univ Melbourne, and (b) HKUST, NUS and NTU. It can be seen that for Caltech, Univ Melbourne and Univ Tsukuba, the CPP difference between their international collaborated publications and their publications without international co-authorship is approximately 4–5. This explains their performance in Figs. 2 and 3: with the increase of international co-authorship rate in their publications, the overall CPP of their papers has more weight from their international co-authored publications, and the overall CPP of their publications are increased. Yet, for HKUST, NUS and NTU, the CPP gaps between publications with and without international co-authorship are relatively small (around 0–1 citations per paper increment). This is because the fact that these institutions have attracted a lot of researchers with international background to work in these institutions. By internalizing the collaborating “external” authors, a special kind of international collaboration has been created that are not expressed in co-authorship, which makes the difference between their national research and international collaborated research relatively small.

**Fig. 5:**
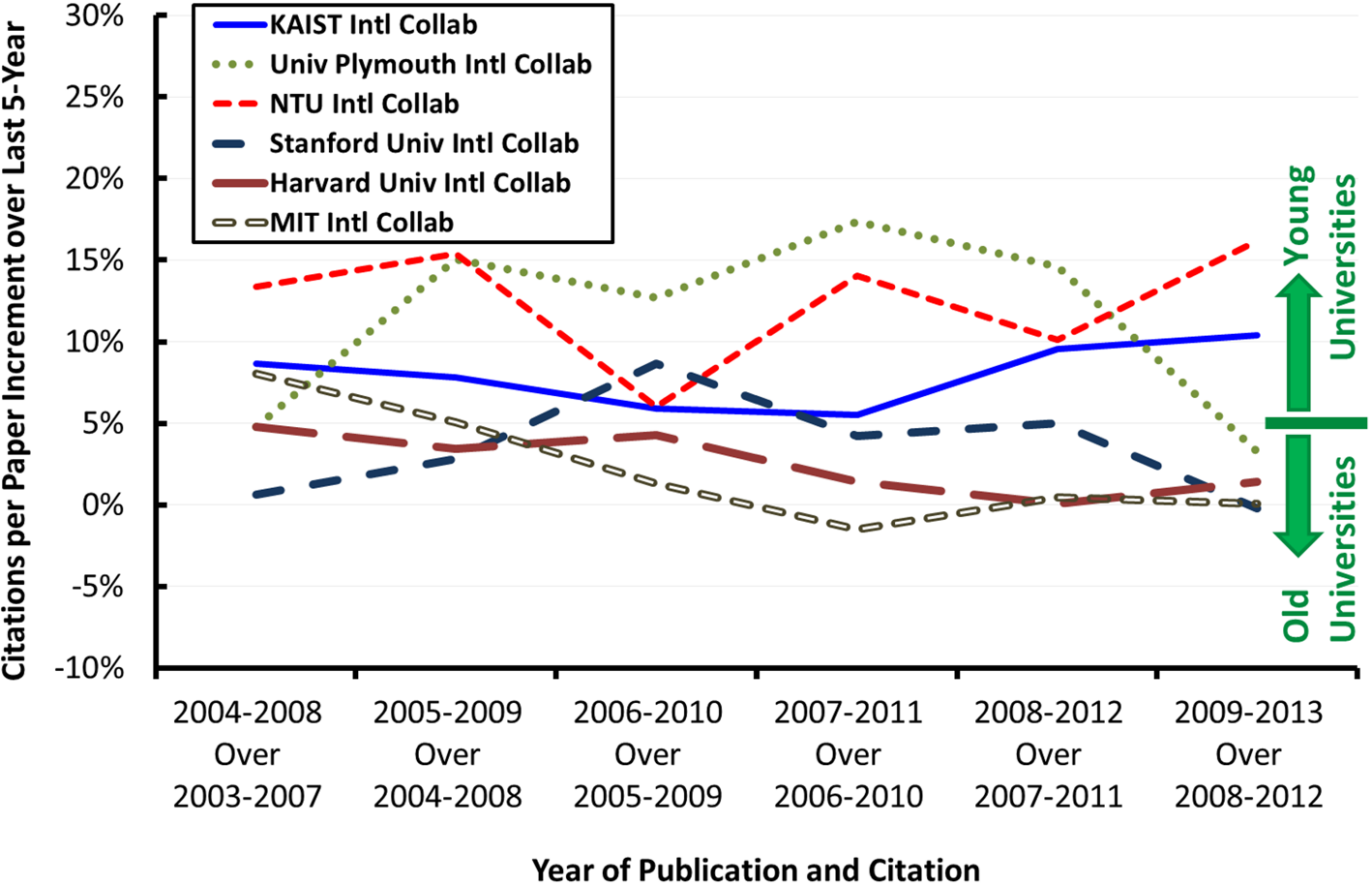
Relative CPP increment over last 5-year period. *KAIST* Korea Advanced Institute of Science and Technology (South Korea), *Univ Plymouth* Plymouth University (UK), *NTU* Nanyang Technological University (Singapore), *Stanford Univ* Stanford University (USA), *Harvard Univ* Harvard University (USA), *MIT* Massachusetts Institute of Technology (USA)

Figure 5 shows the relative CPP increment over the last 5-year period for selected young and old institutions. It can be seen that young institutions has relatively higher relative CPP increment over the last 5-year period compared to that of old institutions.

### Increment of field weighted citation impact (FWCI) of internationally co-authored papers over the FWCI of the involved institutions

**Fig. 6:**
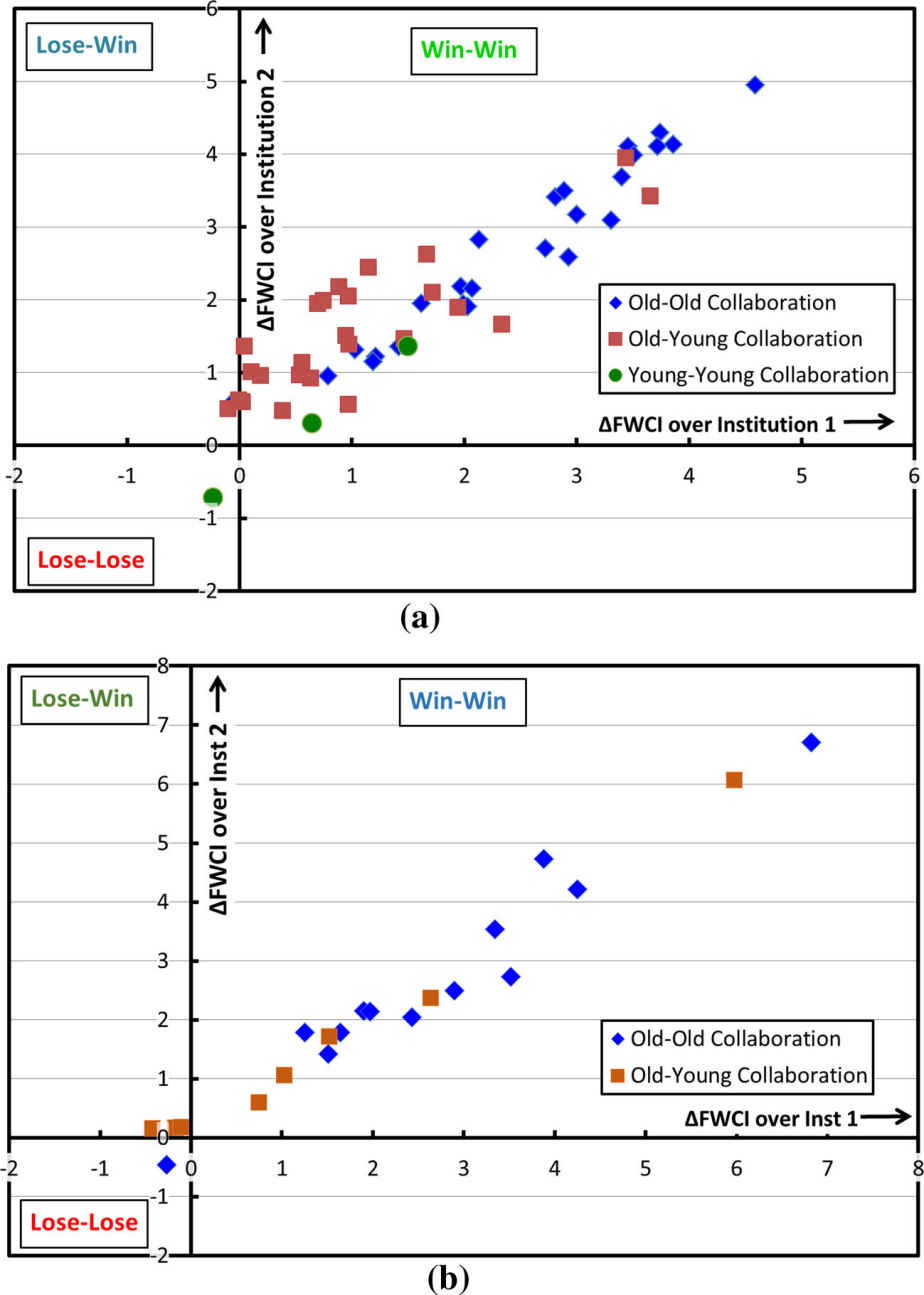
Increment of 5-year FWCI of internationally co-authored papers over the overall FWCI of the involved Institutions. **a** Collaboration between renowned old & young universities, and **b** Collaboration between not so well-known old & young universities

Figure 6 shows (a) the increment of FWCI for internationally co-authored papers over the overall FWCI of the two collaborating institutions among the selected 8 old institutions and 8 young institutions, and (b) the FWCI increment for internationally co-authored papers over the overall FWCI of the two collaborating institutions among the selected “lower ranked” 10 old and 10 young Universities (those in the 200–250 rank universities in the 2014 Times Higher Education WUR).

For the 16 renowned young and old universities, 57 bilateral collaboration couples with 50 and more co-authored publications are identified, and the FWCI increment data for these collaboration couples are include in the plot. The original data for the plot are provided in Tables 1 and 2. Some specific pairs of universities in Tables 1 and 2 showing the largest/lowest impact increase are chosen and the collaboration types are analysed, as shown in Table 3. It can be seen that, international co-authorship benefits both the young and the old institution, with the old institution to old institution collaboration provides the highest FWCI increment, followed by the old institution to young institution collaboration. Young institutions profit from their international collaboration with renowned universities. This may also apply to the national collaboration with renowned local universities. As mentioned in Introduction, internationally collaborated papers will be more visible and more frequently cited in prestigious journals than papers without international collaborations, and so the collaboration with well-known national institutions may also be beneficial to young universities. Among the 57 bilateral collaborations, only 3 involved young institution to young institution collaboration, indicating that there are untapped potential for enhancement on bilateral collaboration among young institutions. From Fig. 6b, we can see that, compared to the renowned universities, those not well-known universities have less collaborations (with 50 and above collaborated publications) among them. There are only 15 old–old collaborations and 6 old–young collaboration found, and there is no collaboration among young to young universities, indicating that there is still large room for the young–young collaborations among the not-so-well-known institutions. It can also be seen from Table 3 that multinational collaboration is the dominant collaboration type for the selected institution pairs, and publications from multinational collaboration have higher citation impact than the publications by binational/bilateral collaborations.

**Table 1:**
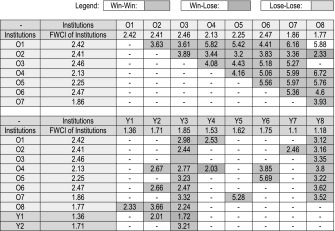
Overall FWCI of the Institutions and FWCI of internationally co-authored papers for selected old & young universities

Selected Institutions: O1: Harvard University; O2: MIT; O3: CalTech; O4: University of Cambridge; O5: University of Oxford; O6: Stanford University; O7: University of Melbourne; O8: National University of Singapore; Y1: City University of Hong Kong; Y2: Hong Kong University of Science and Technology; Y3: Nanyang Technological University; Y4: Pohang University of Science And Technology; Y5: University of East Finland; Y6: Plymouth University; Y7: King Fahd University of Petroleum and Minerals; Y8: University of Tsukuba

**Table 2:**
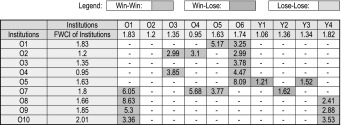
Overall FWCI of the Institutions and FWCI of internationally co-authored papers for selected normal old & young universities

Selected Institutions: O1: University of Goteborg (Sweden); O2: Nagoya University (Japan); O3: SUNY Albany (USA); O4: University of São Paulo (Brazil); O5: University College Dublin (Ireland); O6: University of Oslo (Norway); O7: University of Newcastle (Australia); O8: Georg-August-University Goettingen (Germany); O9: University of Bern (Switzerland); O10: University of Basel (Switzerland); Y1: Dublin Institute of Technology (Ireland); Y2: RMIT University (Australia); Y3: Ulster University (UK); Y4: University of Ulm (Germany); Y5: University of Ulsan (South Korea); Y6: Concordia University (Canada); Y7: City University of London (UK); Y8: Tampere University of Technology (Finland); Y9: Polytechnic University of Valencia (Spain); Y10: National Yang-Ming University (Taiwan)

**Table 3:**
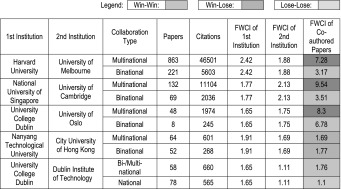
FWCI of the internationally co-authored papers in relation to the collaboration type (binational or multinational) for selected university pairs

Moed (2005b) has done similar pair-wise analysis of incremental impact increase in his chapter “Does scientific collaboration pay?”. He found that when scientifically advanced countries collaborate with one another, they profit in around 7 out of 10 cases from such bi-lateral collaboration, in the sense that both raise their citation impact compared to that of their ‘purely domestic’ papers. But when advanced countries contribute in bilateral international co-authorship to the development of scientifically less advanced countries—and thus to the advancement of science in the longer term than the perspective normally adopted in research evaluation—this activity tends to negatively affect their short-term citation impact, particularly when their role is secondary. As mentioned in Introduction, it would be very interesting if one could exam the “research guarantor” indicator on the international co-authorship of the young institutions. Yet, we will not address this in the present study.

### Correlation between research expenditure in financial year 2013 and 5-Year CPP (2009–2013)

**Fig. 7:**
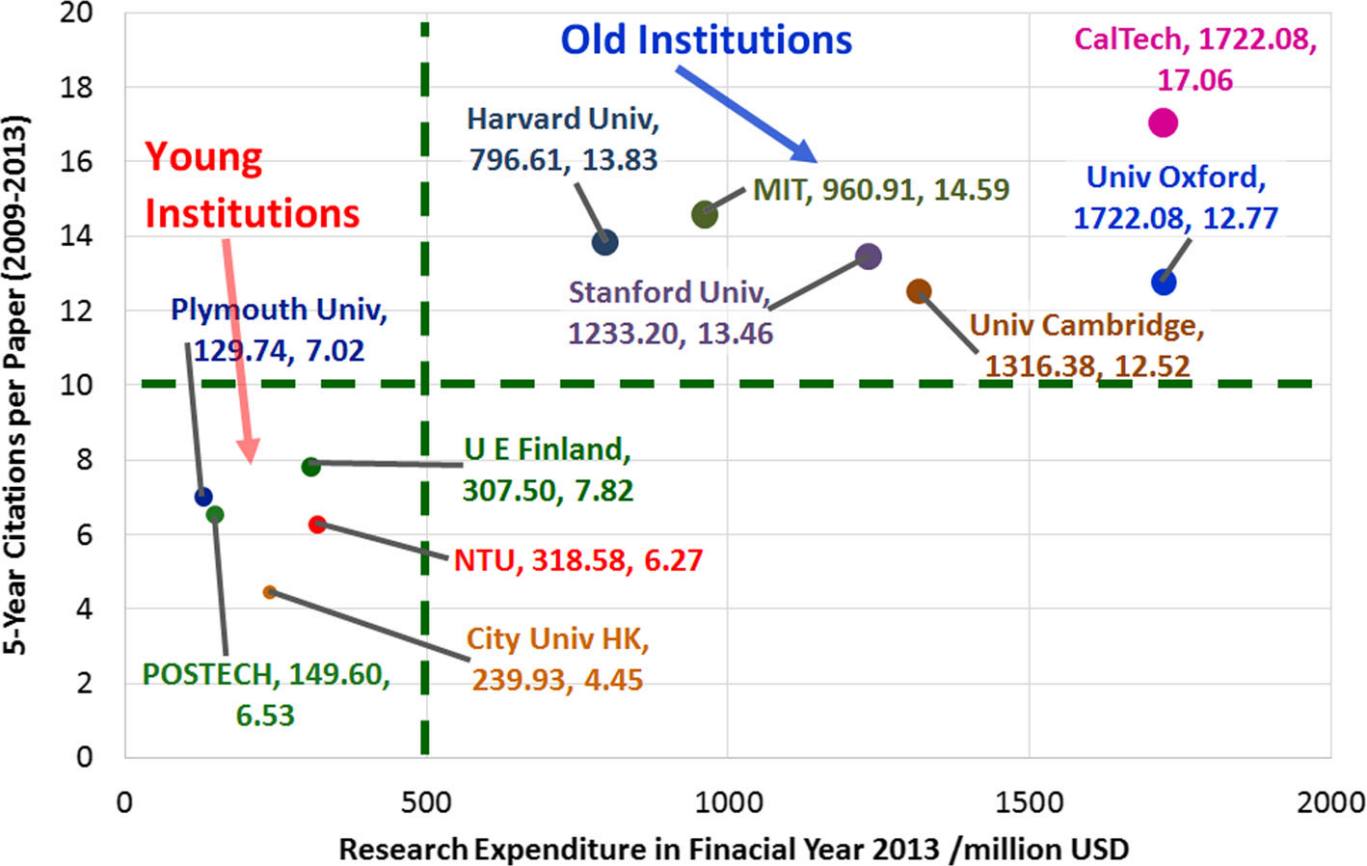
5-year CPP versus research expenditure in 2013

Figure 7 plots the CPP of selected young and old institutions versus the research expenditure in financial year 2013. It can be seen that old institutions has higher research expenditure in financial year 2013 as compared to young institutions.

### Trends of Difference between percentage of international co-authored publications falling in global top 10 % highly cited publications and that for all publications (Δ%_Top10%_)

**Fig. 8:**
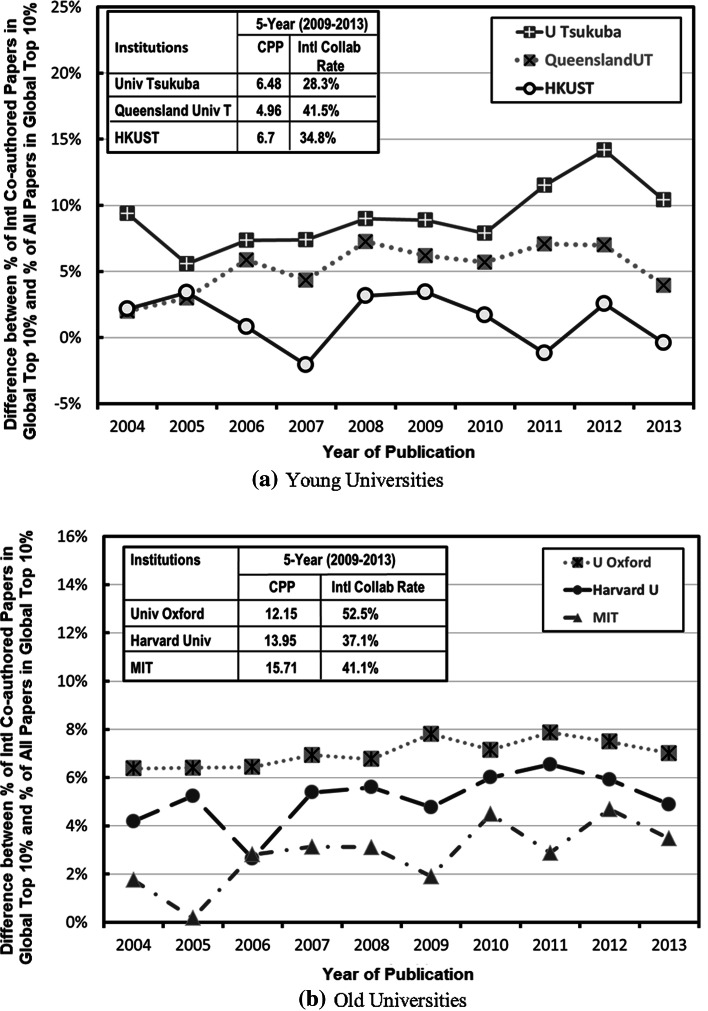
Difference for percentage of publications in global top 10 % highly cited publications for all journal publications and for internationally co-authored publications of the selected **a** young universities, **b** old universities

Figure 8 shows the difference between the percentages of international co-authored publications for an institution falling in the ESI global top 10 % highly cited publications and the percentage of all publications of the same institution falling in the ESI global to top10 % highly cited publications (Δ%_Top10%_). It can be seen that for all the selected young and old institutions, this difference is generally positive, means that internationally collaborated publications generally have a higher rate of high citation publications among all publications. Yet, this difference varies from one institution to another institution. For some renown world top universities like Caltech, Stanford University and University of Cambridge, although their overall CPP for their publications is already very high, the percentage of their internationally collaborated publications falling in the global top 10 % highly cited publications is still higher than the percentage of their overall publications falling in the global top 10 %. Further investigation is needed to have an adequate explanation for this phenomenon.

Table 4 provides the 5-year CPP trends as a function of MGCD. It can be seen that although the old universities has a higher CPP value in general, young institutions have relatively higher CPP increment over MGCD increment.

**Table 4:**
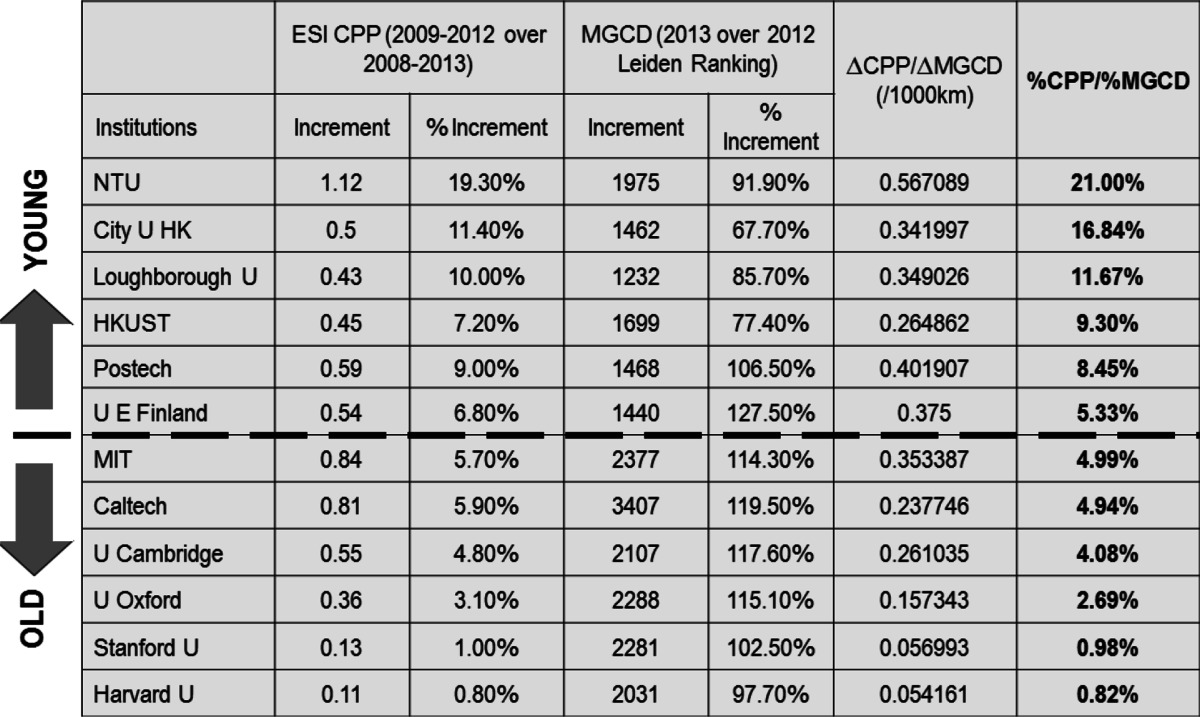
CPP increment with respect of MGCD increment

## Conclusions

In this study, the effect of international co-authorship on the impact of publications of selected young universities and well established renowned universities was investigated. The results show that, both young and old institutions benefitted from international co-authorship as evident by the citation impact of their publications. For example, the CPP increased by 4.12 cites for every 10 % increase in international co-authorship rate for MIT, 3.42 cites for University of Oxford, and 3.01 cites for Stanford University. Among young universities, NTU, the increase is 2.24 CPP per 10 % Intl Collab increment, and for Plymouth University it is 3.02 CPP per 10 % Intl Collab increment, and 0.73 CPP per 10 % Intl Collab increment for King Fahd Univ of Petr and Min. Young universities has a higher relative CPP increment for rolling 5-year measurement in the period 2003–2013.

The percentage of publications in the ESI global top 10 % highly cited publications for international co-authored publications is generally higher than that for all journal publications of the same institution. Yet, this difference varies from one institution to another institution. For some renowned top universities like Caltech, Stanford University and University Cambridge, although their overall CPP for their publications is already very high, the percentage of their internationally collaborated publications falling in the global top 10 % highly cited publications is still higher than the percentage of their overall publications falling in the global top 10 %.

Asian institutions like HKUST, NUS and NTU have attracted a lot of researchers with international background to work in there. As the collaborating “external” authors are internalized in these institutions, a special kind of international collaboration that are not expressed in co-authorship has been created, and the CPP gaps between publications with and without international co-authorship are relatively small (around 0–1 citations per paper increment).

The international co-authorship also increases the FWCI of the institution, yet there are untapped potential to enhance the collaboration among young institutions.

With the increase of international co-authorship ratio, the mean geographical collaboration distance (MGCD) of one institution also increases. Although the old universities has a higher CPP value in general, young institutions have relatively higher CPP increment over MGCD increment.
